# Red and Processed Meat Intake, Polygenic Risk and the Prevalence of Colorectal Neoplasms: Results from a Screening Colonoscopy Population

**DOI:** 10.3390/nu16162609

**Published:** 2024-08-08

**Authors:** Ruojin Fu, Xuechen Chen, Teresa Seum, Michael Hoffmeister, Hermann Brenner

**Affiliations:** 1 Division of Clinical Epidemiology and Aging Research, German Cancer Research Center (DKFZ), Im Neuenheimer Feld 581, 69120 Heidelberg, Germany; 2 Medical Faculty Heidelberg, Heidelberg University, Im Neuenheimer Feld 672, 69120 Heidelberg, Germany; 3 College of Pharmacy, Jinan University, Guangzhou 511436, China; 4 German Cancer Consortium (DKTK), German Cancer Research Center (DKFZ), Im Neuenheimer Feld 280, 69120 Heidelberg, Germany

**Keywords:** colorectal neoplasm, red meat intake, processed meat intake, polygenic risk score, genetic risk equivalent

## Abstract

High red and processed meat intake and genetic predisposition are risk factors of colorectal cancer (CRC). However, evidence of their independent and joint associations on the risk of colorectal neoplasms is limited. We assessed these associations among 4774 men and women undergoing screening colonoscopy. Polygenic risk scores (PRSs) were calculated based on 140 loci related to CRC. We used multiple logistic regression models to evaluate the associations of red and processed meat intake and PRS with the risk of colorectal neoplasms. Adjusted odds ratios (aORs) were translated to genetic risk equivalents (GREs) to compare the strength of the associations with colorectal neoplasm risk of both factors. Compared to ≤1 time/week, processed meat intake >1 time/week was associated with a significantly increased risk of colorectal neoplasm [aOR (95% CI): 1.28 (1.12–1.46)]. This risk increase was equivalent to the risk increase associated with a 19 percentile higher PRS. The association of red meat intake with colorectal neoplasm was weaker and did not reach statistical significance. High processed meat intake and PRS contribute to colorectal neoplasm risk independently. Limiting processed meat intake may offset a substantial proportion of the genetically increased risk of colorectal neoplasms.

## 1. Introduction

Red and processed meat intake is an essential part of daily diet in many societies. However, there is concern about potential adverse health effects of high consumption [1], and the International Agency for Research on Cancer has classified them as carcinogenic [2]. One of the cancers for which high intake of red and processed meat may be of particular concern is colorectal cancer (CRC) [3], the second leading cause of cancer mortality [4]. In most cases, CRC develops slowly from adenoma to carcinoma over a period of 10 or more years, which provides good opportunities for prevention [5,6].

Understanding the interaction between genetic and environmental factors can help unravel the mechanisms behind colorectal carcinogenesis [7], and the joint consideration of these factors may help target individuals with high risk for more effective prevention. Polygenic risk scores (PRSs), derived from genome-wide association studies (GWASs), have proven to be effective in risk prediction of CRC and its precursors, such as advanced adenoma, and identification of people at high genetic risk [8,9,10]. Previous research has investigated the individual and joint associations of red and processed meat intake and PRS with the risk of CRC [11]. However, evidence on the individual and joint associations of red and processed meat intake and polygenic risk with the presence of CRC precursors, which may be highly relevant for risk stratification and targeted prevention, is still very limited.

We aimed to assess the individual and joint association of red and processed meat intake and a polygenic risk score with the presence of colorectal neoplasms in a large population of participants in a CRC screening program. Moreover, we translated the risk of red and processed meat intake into the equivalent effect caused by background genetic risk using the genetic risk equivalent (GRE), a recently established risk communication metric [12].

## 2. Methods

### 2.1. Study Design and Study Population

Our analysis is based on data from the BliTz (Begleitende Evaluierung innovativer Testverfahren zur Darmkrebsfrüherkennung) study. The BliTz study is a large ongoing study aiming at exploring noninvasive approaches for early detection of CRC or its precursors among participants of the German screening colonoscopy program (initiated in 2002, offering screening colonoscopy to men and women aged 55 years or older; the starting age was lowered to 50 for men in 2019). The BliTz study was approved by the Ethics Committees of the Medical Faculty Heidelberg (178/2005), and was registered at the German Clinical Trials Register (DRKS-ID: DRKS00008737). The current analysis was based on data from participants with available environmental and genetic risk factors data who were enrolled from November 2005 to January 2019.

Detailed information on the BliTz study has been described elsewhere [13,14]. In short, since 2005, participants who were interested, willing and capable of cooperating in the study were recruited from 20 gastroenterology clinics in southwestern Germany. Patients were informed and invited to participate in the study during the visit to the practice before the screening colonoscopy. Information on socioeconomic, demographic, lifestyle factors, medical history, and family history of CRC was collected before the colonoscopy using standardized questionnaires. Colonoscopy and histology reports were collected, and findings were extracted independently by two trained data extractors who were blind to questionnaire and genetic data.

### 2.2. Assessment of Red and Processed Meat Intake

In the questionnaires, participants were asked to provide details on their consumption frequency of red meat and processed meat separately in the previous year. Originally, participants were given a range of options to choose from, including never, less than once per week, once per week, multiple times per week, once per day, and multiple times per day. The frequency was first categorized into 2 levels: ≤1 time/week and >1 time/week, and frequency >1 time/week was further divided into 2 levels: >1 time/week and <1 time/day, and ≥1 time/day to evaluate the individual association of processed meat intake and the risk of colorectal neoplasm.

### 2.3. Derivation of Polygenic Risk Score

Genotyping was done by Global Screening Array (Illumina, San Diego, CA, USA) for 3867 participants in 2017 and 2020 and Illumina OncoArray-500 k V1.0 BeadChip (Illumina, San Diego, CA, USA) for 907 participants in 2014. Genotyping was performed for all participants with colorectal neoplasm and available blood samples and random samples of participants without neoplasms who had been recruited by the time of genotyping. A weighted PRS based on 140 loci related to CRC identified in a recent GWAS among 55,105 CRC cases and 65,079 controls of European ancestry [10] was calculated considering both the sum of risk alleles of each variant (0, 1, or 2 copies of the risk allele for genotyped loci; imputed dosages for imputed loci) and the strength of association with CRC risk for each risk allele as shown in their beta coefficients (Table S1). The PRS was further categorized according to the distribution of PRS among participants with no finding in colonoscopy by tertiles.

### 2.4. Statistical Analysis

From all participants recruited by the end of 2019, we excluded those with missing information on red and processed meat intake. According to the aim of the study and to rule out the potential risk introduced into the comparison group, we further excluded participants for whom hyperplastic polyps, non-defined polyps, or serrated adenomas/polyps <1 cm were reported as most advanced finding at colonoscopy. To ensure the representativeness of an average-risk screening population and to minimize the risk of missed neoplasms we furthermore excluded those matching any of the following criteria: age <50 or ≥80 years; history of CRC or inflammatory bowel disease; history of colonoscopy examination in the preceding five years; inadequate bowel preparation; incomplete colonoscopy (coecum not reached). The analysis of the joint association of red/processed meat intake and PRS was further restricted to participants with available genotyping data.

Participants were classified according to the most advanced finding at colonoscopy: any neoplasm [including advanced neoplasm: CRC and advanced precancerous lesions (adenomas with at least one of the characteristics: ≥1 cm in size, tubulovillous or villous components, or high-grade dysplasia; and sessile serrated polyps ≥1 cm in size); and non-advanced adenoma] and no finding. The distribution of characteristics was described and compared between the groups using the Chi-square test for categorical variables. Multiple imputations were then performed for the missing values in relevant factors [years of schooling, body mass index, smoking status, alcohol consumption, physical activity, history of hormone replacement therapy in women, history of diabetes, use of non-steroidal anti-inflammatory drugs (NSAIDs), whole grain intake, fruit intake, vegetable intake, poultry meat intake] using the R package mice 3.7.0 [15].

We first used logistic regression to assess the independent associations of red/processed meat intake with the presence of colorectal neoplasms in models adjusted for age and sex. The models were further adjusted for educational years (<10/10–11/>11 years of schooling), history of CRC in a first-degree relative (yes/no), history of previous colonoscopy (yes/no), smoking status (never/former/current), alcohol consumption (none/0-<12/12–<25/25-<50/≥50 g/day), physical activity (<30 min/day or ≥30 min/day), body mass index (<25/25–<30/≥30 kg/m^2^), diabetes history (yes/no), history of hormone replacement therapy in women (yes/no), use of NSAIDs (yes/no), whole grain intake (<1/≥1 time/day), fruit intake (<1/≥1 time/day), vegetable intake(<1/≥1 time/day) and poultry meat intake(<1/≥1 time/week).

For the joint association of red/processed meat intake and PRS levels, we first analyzed the individual association of red/processed meat intake and PRS levels with the risk of colorectal neoplasm in the corresponding population. PRS was additionally included in the model for the analysis of red/processed meat intake, and red and processed meat intake was included in the model for the analysis of PRS. If significantly increased risks were observed for red/processed meat intake, further analyses were performed in the following order: interaction analysis with PRS, joint evaluation of risk according to both red/processed meat intake and PRS, and relevant GRE calculation. Interaction analyses were conducted by including a cross-product term of PRS and red/processed meat intake along with the main effect terms in multivariable regression models. The joint associations of red/processed meat intake and PRS were assessed using meat intake ≤1 time/week with lowest PRS tertile as the uni-reference.

Adjusted odds ratios (aORs) of red/processed meat intake were translated into the GREs for risk communication if the association reached statistical significance. GRE is a metric that was recently developed based on the well-established concept of risk and rate advancement period [16]. Details on the derivation of GREs are provided in the Supplement Method. In brief, GREs were calculated as the ratios of regression coefficients, obtained from logistic regression models, for red/processed meat intake and PRS percentiles. They quantify how much of the genetic risk (calculated as the percentiles of the PRS) may be “compensated for” by avoiding the investigated risk factors. For example, a GRE of 30 means that avoiding a specific risk factor may have an equivalent effect as having a 30 percentile lower PRS.

All statistical analyses were performed by R, version 4.1.3 (R Foundation for Statistical Computing, Vienna, Austria). Statistical significance was defined as two-sided and *p* < 0.05.

## 3. Results

### 3.1. Characteristics of the Study Population

**Figure 1:**
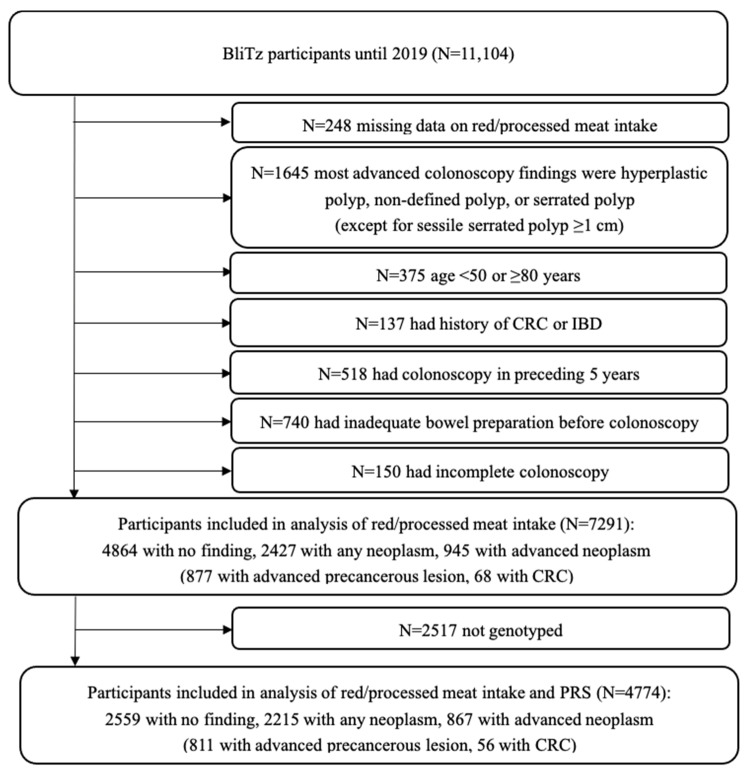
Flowchart of inclusion of study participants. Abbreviations: CRC, colorectal cancer; IBD, inflammatory bowel disease; PRS, polygenic risk score.

A total of 7291 participants (2427 with colorectal neoplasm, including 877 with advanced precancerous lesions and 68 with CRC, and 4864 without colorectal neoplasm) with complete information on red and processed meat intake were included for the evaluation of the individual association of red and processed meat intake with the prevalence of colorectal neoplasms. For the joint consideration of red and processed meat intake and PRS, with the exclusion of participants without genotyping information, 4774 participants were included (2215 with colorectal neoplasm, including 811 with advanced precancerous lesions and 56 with CRC, and 2559 without colorectal neoplasm) (Figure 1).

The baseline characteristics of the study populations are shown in Table S2 (for the participants with complete information on red and processed meat only) and Table 1 (for the participants with complete information on red and processed meat and PRS). 

### 3.2. Associations between Red and Processed Meat Intake, PRS, and Prevalence of Colorectal Neoplasms

The individual association of red/processed meat and colorectal neoplasm risk was first evaluated in the entire population with complete information on red and processed meat, including participants who had not been genotyped (Table S3). Among 7291 participants, processed meat intake >1 time/week was associated with an increased risk of carrying a colorectal neoplasm [aOR (95% CI): 1.28(1.12, 1.46)], whereas no statistically significant association was observed for red meat intake [aOR (95% CI): 1.05(0.95, 1.17)]. The corresponding risks for advanced neoplasm were similar to those of any colorectal neoplasm. No dose-response relationship was found when further separating processed meat intake >1 time/week into >1 time/week and <1 time/day, and ≥1 time/day.

In the study population with both questionnaire and genotype data, very similar results were obtained on the individual associations of red and processed meat intake and the prevalence of colorectal neoplasms (Table 2). 

Furthermore, PRS was strongly related to the prevalence of colorectal neoplasms. Compared to participants in the lowest PRS tertile, those in the highest tertile had an almost two-fold increased risk (aOR 1.95, 95% CI 1.68, 2.26). With an aOR of 2.47 (95% CI 2.00, 3.04), the association with advanced neoplasms was even stronger (Table 3).

Since there was no statistically significant association between red meat intake and the risk of carrying colorectal neoplasm, further analyses in conjunction with PRS were only performed for processed meat intake. These analyses did not show any statistically significant interaction of processed meat intake with PRS levels. A strong dose-response relationship of PRS with the risk of any advanced colorectal neoplasm or any advanced colorectal neoplasm was seen regardless of the frequency of processed meat consumption (Table 4). Compared to people consuming processed meat ≤1 time/week and a PRS in the lowest tertile, those with more frequent processed meat consumption and with highest PRS tertile had a 2.3-fold risk of carrying any neoplasm and a 3.8-fold risk of carrying an advanced colorectal neoplasm (Table 5).

### 3.3. Genetic Risk Equivalents for High Frequency of Processed Meat Intake

The estimates of GRE for processed meat intake are shown in Table 6. Processed meat intake >1 time/week was associated with an equivalent increase in risk of carrying any colorectal neoplasm as having a 19.0% [GRE (95% CI): 19.0 (3.2, 34.7)] higher PRS. The GRE estimate for carrying an advanced colorectal neoplasm was slightly lower, given the stronger association of the PRS with this outcome.

## 4. Discussion

In this large screening colonoscopy study, we found that a high frequency of processed meat intake and higher PRS levels were independently associated with an elevated risk of carrying colorectal neoplasm. When considering processed meat intake and PRS together, individuals in the highest tertile of PRS consuming processed meat >1 time/week had an approximately 2.3- and 3.8-fold increased risk of carrying any neoplasms and advanced neoplasm when compared to participants in the lowest PRS tertile consuming processed meat ≤1 time/week. Our GRE estimate indicates that the risk increase associated with consumption of processed meat intake >1 time/week corresponds to that of having a 19 percentile higher level of PRS, underlining the importance of lowering the frequency of processed meat intake from a novel perspective of equivalent polygenic risk.

Meat is a major source of proteins, minerals, and vitamins in nutrition of mankind [17]. However, the International Agency for Research on Cancer classified red meat as likely to cause cancer (Group 2A) and processed meat as carcinogenic (Group 1) for human beings [2]. There are several potential carcinogenesis mechanisms, including the formation of carcinogenic heterocyclic amines (HCAs) and polycyclic aromatic hydrocarbons (PAHs) during high-temperature cooking, the heme iron content of red meat, the nitrate and nitrite content of processed meat, and adverse alterations of the gut microbiome [18]. Although there is ongoing debate to what extent the intake of red and processed meat should be limited [19,20], evidence has been emerging that high consumption is related to several major diseases, such as cardiovascular disease, diabetes and colorectal cancer [21]. However, evidence on the association of red and processed meat intake with the risk of colorectal adenomas has been limited. Although results have not been entirely consistent, a dose–response meta-analysis published in 2013 showed a 36% (95% CI 17% to 58%) increased risk of colorectal adenoma per 100 g/day higher red meat intake, and a 28% (95% CI 3% to 60%) increased risk of colorectal adenoma per 50 g/day higher processed meat intake [22]. In a study from another large screening colonoscopy cohort from Germany published in 2017, processed meat was positively associated with the prevalence of advanced adenomas in the rectum only [23]. An updated systematic review and meta-analysis published in 2018 concluded that red and processed meat consumption was associated with increased risk of colorectal neoplasms, but not with the risk of recurrence [24]. A most recent study including over 1000 participants, which was also based on the screening setting, reported higher consumption of processed meat or the combination of processed and red meat, but not higher consumption of red meat intake alone, was associated with a significantly increased risk of advanced adenoma [25]. Our findings from a much larger study population support the evidence that an increased risk is mainly associated with high consumption of processed meat.

As far as we know, we are the first to examine the joint associations of processed meat consumption and PRS with the risk of carrying colorectal neoplasms. Our results provide evidence for the notion that incorporating both PRS and lifestyle factors may enhance the ability to identify high-risk individuals of developing colorectal neoplasms for risk-adapted CRC screening. The calculation of GREs may be a useful tool to facilitate risk communication in this context. To our knowledge, our study is the first to report GREs of high processed meat intake for colorectal neoplasms including both colorectal adenomas and CRC. The GREs for colorectal neoplasms indicate that the estimated impact of high processed meat consumption is equivalent to the effect of having a substantially higher PRS. In simpler terms, our results suggest that a substantial share of genetically increased risk of colorectal neoplasms might be compensated for by reducing the frequency of processed meat intake. GRE is a clear and easily comprehensible quantitative measure, which, in studies on CRC risk, has been shown to have the potential to assist the public in grasping the impact of unhealthy lifestyle choices and promoting conformity to health guidelines [12,26]. Its use in assessing cancer precursor risk, as in our study, could further aid in increasing awareness for cancer prevention through lifestyle changes.

There are several strengths of our study. The BliTz study is among the largest colorectal screening studies worldwide with genetic and lifestyle risk factor data collected ahead of colonoscopy. The study was performed in a screening colonoscopy population which, unlike study populations from many studies conducted in clinical settings, well represents the target population for CRC screening. Our study considered both genetic risk and processed meat intake to identify those who are at a high risk of carrying colorectal neoplasms, which could enhance the risk stratification. As far as we know, this is the first study to quantify the relationship between processed meat intake and the risk of colorectal neoplasm through defined variations in PRS levels using the recently established GRE metric.

There are also several limitations. First, accuracy of information on red and processed meat consumption may have been affected by imperfect recall and reporting. Second, only frequency, but not the amount of meat intake was asked for in the questionnaire. Third, despite the relatively large size of the study, some associations were not statistically significant, and no dose-response relationship was observed for processed meat which may be caused by random variation given the limited sample size and lack of detailed information of processed meat intake other than the frequencies. Studies with larger sample sizes and more detailed information such as the quantities and types of the processed meat intake should further explore these associations in the future. Fourth, residual confounding resulting from incomplete recollection or unaccounted risk factors cannot be entirely ruled out, even though many covariates were taken into consideration when adjusting the statistical models. Fifth, the PRS was built using a limited set of the 140 CRC-related loci identified for the population of European ancestry. Further developments of PRS with additional loci and more sophisticated approaches of calculation might more accurately predict the risk of colorectal neoplasia. Lastly, the generalizability to other populations of the findings is limited due to the ethnic homogeneity of the study population, which consisted primarily of white residents in southwest Germany.

## 5. Conclusions

Our study contributes to the evidence of the relationship between red and processed meat intake, PRS and the prevalence of colorectal neoplasms. Our results suggest that less frequent consumption of processed meat intake might have the potential to compensate for a substantial share of genetically increased risk. Future research should follow-up these findings by more precise quantification of the amount and type of processed meat consumption and genetic risk in diverse populations. We hope that our results may be helpful to inform efforts of CRC prevention and contribute to enhanced risk assessment to inform risk-adapted CRC screening strategies.

## Figures and Tables

**Table 1:** Characteristics of the study population with complete information on both red and processed meat intake and polygenic risk score.

Characteristics	No Finding, N (%)	Any Neoplasm, N (%)		*p*-Value ^f^
		Overall	Advanced Neoplasm	
Total	2559	2215	867	
Sex				<0.0001
Female	1436 (56.1%)	830 (37.5%)	333 (38.4%)	
Male	1123 (43.9%)	1385 (62.5%)	534 (61.6%)	
Age (year, Median (Q^25^, Q^75^))	60 (56, 66)	62 (57, 68)	62 (57, 68.5)	<0.0001
Education (year)				0.0023
<10	1297 (50.7%)	1227 (55.4%)	479 (55.2%)	
10–11	659 (25.8%)	532 (24.0%)	193 (22.3%)	
>11	581 (22.7%)	432 (19.5%)	184 (21.2%)	
BMI (kg/m^2^)				<0.0001
<25	963 (37.6%)	625 (28.2%)	260 (30.0%)	
25–<30	1047 (40.9%)	1034 (46.7%)	387 (44.6%)	
≥30	505 (19.7%)	526 (23.7%)	209 (24.1%)	
Smoking status				<0.0001
Never	1304 (51.0%)	937 (42.3%)	336 (38.8%)	
Former	921 (36.0%)	842 (38.0%)	324 (37.4%)	
Current	304 (11.9%)	417 (18.8%)	201 (23.2%)	
PRS ^a^				<0.0001
T1	853 (33.3%)	521 (23.5%)	179 (20.6%)	
T2	853 (33.3%)	690 (31.2%)	263 (30.3%)	
T3	853 (33.3%)	1004 (45.3%)	425 (49.0%)	
Alcohol consumption ^b^				<0.0001
None	646 (25.2%)	459 (20.7%)	188 (21.7%)	
Low	954 (37.3%)	752 (34.0%)	264 (30.4%)	
Low-moderate	497 (19.4%)	509 (23.0%)	199 (23.0%)	
Moderate-high	282 (11.0%)	321 (14.5%)	143 (16.5%)	
High	77 (3.0%)	96 (4.3%)	46 (5.3%)	
Physical activity ^c^				0.7066
<30 min/day	74 (2.9%)	69 (3.1%)	26 (3.0%)	
≥30 min/day	2451 (95.8%)	2113 (95.4%)	829 (95.6%)	
Red meat intake ^d^				0.0001
≤1 time/week	1193 (46.6%)	897 (40.5%)	353 (40.7%)	
>1 time/week	1366 (53.4%)	1318 (59.5%)	514 (59.3%)	
Processed meat intake ^d^				<0.0001
≤1 time/week	597 (23.3%)	359 (16.2%)	142 (16.4%)	
>1 time/week	1962 (76.7%)	1856 (83.8%)	725 (83.6%)	
* >1 time/week and <1 time/day*	999 (39.0%)	919 (41.5%)	375 (43.3%)	
* ≥* *1 time/day*	963 (37.6%)	937 (42.3%)	350 (40.4%)	
History of HRT ^e^	538 (21.0%)	297 (13.4%)	111 (12.8%)	0.4804
History of diabetes	221 (8.6%)	269 (12.1%)	108 (12.5%)	0.0001
Family history of CRC	308 (12.0%)	298 (13.5%)	122 (14.1%)	0.1545
Use of NSAIDs	431 (16.8%)	379 (17.1%)	145 (16.7%)	0.7942
History of colonoscopy	824 (32.2%)	566 (25.6%)	189 (21.8%)	<0.0001
Whole grain intake ^d^ (<1 time/day)	1446 (56.5%)	1340 (60.5%)	525 (60.6%)	0.0041
Fruit intake ^d^ (<1 time/day)	970 (37.9%)	909 (41.0%)	372 (42.9%)	0.0362
Vegetable intake ^d^ (<1 time/day)	1180 (46.1%)	1116 (50.4%)	451 (52.0%)	0.0036
Poultry meat intake ^d^ (<1 time/week)	1022 (39.9%)	881 (39.8%)	353 (40.7%)	1.0000

Note: number of missing participants in No finding/Any neoplasm/Advanced neoplasm: education 22/24/11, BMI 44/30/11, smoking status 30/19/6, alcohol consumption 103/78/27, physical activity 34/33/12, history of hormone replacement therapy 20/14/7, history of diabetes 21/7/1, use of NSAIDs 177/160/62, whole grain intake 38/37/22, fruit intake 15/6/5, vegetable intake 5/4/2, poultry meat intake 36/41/13. ^a^ PRS was categorized according to the distribution of PRS among participants with no finding in colonoscopy by tertiles. ^b^ Alcoholic consumption in the past 12 months: None: 0 g/day; Low: 0–<12 g/day; Low-moderate: 12–<25 g/day; Moderate-high: 25–<50 g/day; High: ≥50 g/day. ^c^ Physical activity in the previous 12 months. ^d^ Consumption in the previous 12 months. ^e^ N (%) was calculated among female participants. ^f^ Comparing participants with and without neoplasm. Abbreviations: BMI, body mass index; CRC, colorectal cancer; HRT, hormone replacement therapy; NSAID, non-steroidal anti-inflammatory drug; PRS, polygenic risk score; T, tertile.

**Table 2:** Association of red and processed meat intake with colorectal neoplasms risk among genotyped participants.

	Compared Groups, N (%) ^a^		OR (95% CI) ^b^	OR (95% CI) ^c^
Red meat intake				
	No finding	Any neoplasm		
≤1 time/week	1193 (46.6%)	897 (40.5%)	Ref.	Ref.
>1 time/week	1366 (53.4%)	1318 (59.5%)	1.11 (0.98, 1.25)	1.07 (0.94, 1.21)
	No finding	Advanced neoplasm		
≤1 time/week	1193 (46.6%)	353 (40.7%)	Ref.	Ref.
>1 time/week	1366 (53.4%)	514 (59.3%)	1.12 (0.95, 1.32)	1.07 (0.90, 1.28)
Processed meat intake				
	No finding	Any neoplasm		
≤1 time/week	597 (23.3%)	359 (16.2%)	Ref.	Ref.
>1 time/week	1962 (76.7%)	1856 (83.8%)	1.32 (1.13, 1.53)	1.22 (1.04, 1.43)
* >1 time/week and <1 time/day*	999 (39.0%)	919 (41.5%)	1.34 (1.13, 1.57)	1.26 (1.07, 1.50)
* ≥* *1 time/day*	963 (37.6%)	937 (42.3%)	1.29 (1.10, 1.53)	1.17 (0.98, 1.39)
	No finding	Advanced neoplasm		
≤1 time/week	597 (23.3%)	142 (16.4%)	Ref.	Ref.
>1 time/week	1962 (76.7%)	725 (83.6%)	1.33 (1.08, 1.64)	1.23 (0.99, 1.54)
* >1 time/week and <1 time/day*	999 (39.0%)	375 (43.3%)	1.40 (1.12, 1.75)	1.33 (1.05, 1.68)
* ≥* *1 time/day*	963 (37.6%)	350 (40.4%)	1.25 (1.00, 1.58)	1.12 (0.88, 1.43)

^a^ Participants with complete information on both red/processed meat intake and PRS. ^b^ Adjusted for age and sex. ^c^ Additionally adjusted for education, smoking status, BMI, physical activity, alcohol consumption, history of hormone replacement therapy, history of diabetes, use of NSAIDs, family history of CRC in a first-degree relative, history of colonoscopy, whole grain intake, fruit intake, vegetable intake, poultry meat intake and PRS. Abbreviations: BMI, body mass index; CI, confidence interval; NSAID, non-steroidal anti-inflammatory drug; OR, odds ratio; PRS, polygenic risk score; Ref., reference.

**Table 3:** Association of polygenic risk score with colorectal neoplasms risk.

PRS ^a^	Compared Groups, N (%)		OR (95% CI) ^b^	OR (95% CI) ^c^
	No finding	Any neoplasm		
T1	853 (33.3%)	521 (23.5%)	Ref.	Ref.
T2	853 (33.3%)	690 (31.2%)	1.30 (1.12, 1.52)	1.30 (1.12, 1.52)
T3	853 (33.3%)	1004 (45.3%)	1.94 (1.68, 2.25)	1.95 (1.68, 2.26)
Per tertile			1.40 (1.30, 1.51)	1.40 (1.30, 1.51)
	No finding	Advanced neoplasm		
T1	853 (33.3%)	179 (20.6%)	Ref.	Ref.
T2	853 (33.3%)	263 (30.3%)	1.47 (1.18, 1.82)	1.46 (1.17, 1.82)
T3	853 (33.3%)	425 (49.0%)	2.42 (1.98, 2.97)	2.47 (2.00, 3.04)
Per tertile			1.57 (1.42, 1.73)	1.58 (1.43, 1.76)

^a^ PRS was categorized by tertile according to the distribution of PRS among participants without neoplasms. ^b^ Adjusted for age and sex. ^c^ Additionally adjusted for education, smoking status, BMI, physical activity, alcohol consumption, history of hormone replacement therapy, history of diabetes, use of NSAIDs, family history of CRC in a first-degree relative, history of colonoscopy, whole grain intake, fruit intake, vegetable intake, poultry meat intake and red/processed meat intake. Abbreviations: BMI, body mass index; CI, confidence interval; NSAID, non-steroidal anti-inflammatory drug; OR, odds ratio; PRS, polygenic risk score; Ref., reference; T, tertile.

**Table 4:** Joint association of processed meat intake and polygenic risk score with colorectal neoplasms risk.

Processed Meat Intake	PRS ^a^	Compared Groups, N (%)		OR (95% CI) ^b^	OR (95% CI) ^c^
		No finding	Any neoplasm		
≤1 time/week	T1	197 (33.0)	87 (24.2)	Ref.	Ref.
	T2	191 (32.0)	115 (32.0)	1.32 (0.93, 1.86)	1.34 (0.93, 1.91)
	T3	209 (35.0)	157 (43.7)	1.69 (1.22, 2.36)	1.75 (1.25, 2.46)
	Per tertile			1.30 (1.10, 1.53)	1.32 (1.12, 1.57)
>1 time/week	T1	654 (33.4)	434 (23.4)	Ref.	Ref.
	T2	662 (33.7)	575 (31.0)	1.30 (1.10, 1.54)	1.29 (1.09, 1.54)
	T3	644 (32.8)	847 (45.6)	2.01 (1.71, 2.37)	2.01 (1.70, 2.37)
	Per tertile			1.43 (1.31, 1.55)	1.42 (1.31, 1.55)
*p* value for interaction with PRS ^d^ =				0.54	0.64
		No finding	Advanced neoplasm		
≤1 time/week	T1	197 (33.0)	23 (16.2)	Ref.	Ref.
	T2	191 (32.0)	47 (33.1)	2.05 (1.19, 3.51)	2.10 (1.20, 3.69)
	T3	209 (35.0)	72 (50.7)	2.96 (1.77, 4.93)	3.14 (1.85, 5.35)
	Per tertile			1.67 (1.31, 2.13)	1.73 (1.34, 2.22)
>1 time/week	T1	656 (33.4)	156 (21.5)	Ref.	Ref.
	T2	662 (33.7)	216 (29.8)	1.39 (1.09, 1.76)	1.36 (1.07, 1.74)
	T3	644 (32.8)	353 (48.7)	2.36 (1.89, 2.94)	2.36 (1.88, 2.97)
	Per tertile			1.55 (1.39, 1.73)	1.56 (1.39, 1.74)
*p* value for interaction with PRS ^d^ =				0.45	0.40

^a^ PRS was categorized by tertiles according to the distribution of PRS among participants without neoplasms. ^b^ Adjusted for age and sex. ^c^ Additionally adjusted for education, smoking status, physical activity, alcohol consumption, BMI, history of hormone replacement therapy, history of diabetes, use of NSAIDs, family history of CRC in a first-degree relative, history of colonoscopy, whole grain intake, fruit intake, vegetable intake and poultry meat intake. ^d^ Interaction was tested by additionally including a cross-product term of processed meat intake and PRS in multivariable models. Abbreviations: BMI, body mass index; CI, confidence interval; CRC, colorectal cancer; NSAID, non-steroidal anti-inflammatory drug; OR, odds ratio; PRS, polygenic risk score; Ref., reference; T, tertile.

**Table 5:** Odds ratios (95% confidence intervals) of joint association of processed meat intake and polygenic risk score with colorectal neoplasms risk, using meat intake ≤1 time/week in the lowest PRS tertile as reference.

Processed Meat Intake	Polygenic Risk Score		
	T1	T2	T3
Any neoplasm vs. No finding			
≤1 time/week	Ref.	1.32 (0.93, 1.89)	1.75 (1.25, 2.45)
>1 time/week	1.16 (0.87, 1.56)	1.51 (1.13, 2.01)	2.33 (1.75, 3.09)
Advanced neoplasm vs. No finding			
≤1 time/week	Ref.	2.05 (1.18, 3.57)	3.13 (1.86, 5.29)
>1 time/week	1.61 (0.99, 2.60)	2.19 (1.36, 3.51)	3.79 (2.38, 6.04)

Note: PRS was categorized by tertile according to the distribution of PRS among participants without neoplasms. Odds ratios were adjusted for age, sex, education, smoking status, physical activity, alcohol consumption, BMI, history of hormone replacement therapy, history of diabetes, use of NSAIDs, family history of CRC in a first-degree relative, history of colonoscopy, whole grain intake, fruit intake, vegetable intake and poultry meat intake. Abbreviations: BMI, body mass index; CI, confidence interval; CRC, colorectal cancer; NSAID, non-steroidal anti-inflammatory drug; OR, odds ratio; PRS, polygenic risk score; Ref., reference; T, tertile.

**Table 6:** Genetic risk equivalent for comparisons between processed meat intake frequencies.

Frequency	Compared Groups, N (%)		OR (95% CI) ^a^	GRE (95% CI)
	No finding	Any neoplasm		
≤1 time/week	597 (23.3%)	359 (16.2%)	Ref.	Ref.
>1 time/week	1962 (76.7%)	1856 (83.8%)	1.22 (1.04, 1.42)	19.0 (3.2, 34.7)
	No finding	Advanced neoplasm		
≤1 time/week	597 (23.3%)	142 (16.4%)	Ref.	Ref.
>1 time/week	1962 (76.7%)	725 (83.6%)	1.22 (0.98, 1.52)	14.1 (−1.8, 30.0)

^a^ Adjusted for age, sex, education, smoking status, physical activity, alcohol consumption, BMI, history of hormone replacement therapy, history of diabetes, use of NSAIDs, family history of CRC in a first-degree relative, history of colonoscopy, whole grain intake, fruit intake, vegetable intake, poultry meat intake and PRS. Abbreviations: BMI, body mass index; CI, confidence interval; CRC, colorectal cancer; GRE, genetic risk equivalent; NSAID, non-steroidal anti-inflammatory drug; OR, odds ratio; PRS, polygenic risk score; Ref., reference.

## Data Availability

The data presented in this study are not available due to privacy reasons.

## Supplementary Materials

The following supporting information can be downloaded at: https://www.mdpi.com/article/10.3390/nu16162609/s1, Table S1: Single-nucleotide polymorphisms considered to generate the polygenic risk score; Table S2: Characteristics of the study population with complete information on red and processed meat intake; Table S3: Association of red and processed meat intake with colorectal neoplasms risk among participants with complete information on meat intake; Supplementary method: Derivation of Genetic Risk Equivalent. References [10,16] are cited in the Supplementary Materials.

## Abbreviations

BMI, body mass index; CRC, colorectal cancer; CI, confidence interval; GRE, genetic risk equivalent; GWAS, genome-wide association study; HRT, hormone replacement therapy; IBD, inflammatory bowel disease; NSAID, non-steroidal anti-inflammatory drug; OR, odds ratio; PRS, polygenic risk score; Ref., reference; SNP, single nucleotide polymorphism; T, tertile.
