# Supplementary material for: Red and Processed Meat Intake, Polygenic Risk and the Prevalence of Colorectal Neoplasms: Results from a Screening Colonoscopy Population
Source: Nutrients. 2024 Aug 8;16(16):2609. doi: 10.3390/nu16162609 (PMC11357662; doi:10.3390/nu16162609)
Supplement: Supplementary file 1 [file nutrients-16-02609-s001.zip › nutrients-3137696-supplementary.pdf]

Supplementary Materials

Table S1. Single-nucleotide polymorphisms considered to generate the polygenic risk score (PRS), adapted from Thomas et al. (1)

Locus	rsID lead variant	Variant	Chr.	Position (Build 37)	Risk allele	Reported beta coefficient risk allele
1p34.3	rs4360494	1:38455891_G/C	1	38455891	G	0.0379
1p32.3	rs12144319	1:55246035_T/C	1	55246035	C	0.0661
1p36.12	rs72647484	1:22587728_T/C	1	22587728	T	0.0504
1p31.3	rs7542665	1:62673037_T/C	1	62673037	C	0.0334
1q25.3	rs6678517	1:183002639_A/G	1	183002639	A	0.0730
1q41	rs17011141	1:222112634_A/G	1	222112634	G	0.0877
2q24.2	rs448513	2:159964552_T/C	2	159964552	C	0.0054
2q33.1	rs11884596	2:199612407_T/C	2	199612407	C	0.0342
2q33.1	rs983402	2:199781586_T/C	2	199781586	T	0.0622
2p16.3	rs7606562	2:48686695_T/A	2	48686695	T	0.0414
2q11.2	rs11692435	2:98275354_G/A	2	98275354	G	0.0492
2q35	rs3731861	2:219191256_T/C	2	219191256	T	0.0613
3q22.2	rs10049390	3:133701119_G/A	3	133701119	A	0.0455
3q13.2	rs13086367	3:112903888_A/G	3	112903888	A	0.0463
3q13.2	rs72942485	3:112999560_G/A	3	112999560	G	0.0545
3p21.1	rs9831861	3:53088285_T/G	3	53088285	G	0.0294
3p22.1	rs35470271	3:40915239_A/G	3	40915239	G	0.0994
3q13.2	rs12635946	3:112916918_C/T	3	112916918	C	0.0334
3q22.2	rs113569514	3:133748789_T/C	3	133748789	T	0.0414
3q26.2	rs9876206	3:169517436_C/T	3	169517436	C	0.0453
3p14.1	rs6781752	3:66365163_G/A	3	66365163	A	0.0597
4q31.21	rs11727676	4:145659064_T/C	4	145659064	C	0.0093
4q24	rs1391441	4:106128760_G/A	4	106128760	A	0.0148
4q22.2	rs13149359	4:94938618_C/A	4	94938618	A	0.0520
5p13.1	rs7708610	5:40102443_G/A	5	40102443	A	0.0384
5p15.33	rs78368589	5:1240204_C/T	5	1240204	T	0.0786
5q21.1	rs145364999	5:98206082_T/A	5	98206082	T	0.3496
5p15.33	rs2735940	5:1296486_A/G	5	1296486	G	0.0865
5p13.1	rs12514517	5:40280076_G/A	5	40280076	A	0.1013
5q22.2	rs755229494	5:112097351_A/G	5	112097351	G	0.6286
5q23.2	rs12659017	5:125988175_G/A	5	125988175	G	0.0374
5q31.1	rs4976270	5:134467220_C/T	5	134467220	C	0.0693

Table S1, continued.

Locus	rsID lead variant	Variant	Chr.	Position (Build 37)	Risk allele	Reported beta coefficient risk allele
6p12.1	rs13204733	6:55566108_A/G	6	55566108	G	0.0643
6p21.33	rs116685461	6:31315512_G/A	6	31315512	G	0.0655
6p21.32	rs9271695	6:32593080_A/G	6	32593080	G	0.0889
6p21.33	rs2516420	6:31449620_C/T	6	31449620	C	0.1091
6p21.33	rs116353863	6:31010185_T/C	6	31010185	C	0.1202
6p21.31	rs16878812	6:35569562_A/G	6	35569562	A	0.0778
6p21.2	rs9470361	6:36623379_G/A	6	36623379	A	0.0540
6p12.1	rs62404966	6:55712124_C/T	6	55712124	C	0.0724
6p21.33	rs3131043	6:30758466_A/G	6	30758466	G	0.0294
6p24.1	rs2070699	6:12292772_G/T	6	12292772	T	0.0294
6p22.1	rs1476570	6:29809860_G/A	6	29809860	A	0.0492
6p21.32	rs3830041	6:32191339_C/T	6	32191339	T	0.0645
6q21	rs6928864[a]	6:105966894_C/A	6	105966894	C	0.0531
6p21.1	rs62396735	6:41702582_C/T	6	41702582	C	0.0330
7p13	rs12672022	7:45136423_T/C	7	45136423	T	0.0067
7p12.3	rs80077929	7:46094089_C/T	7	46094089	T	0.0093
7p12.3	rs10951878	7:46926695_C/T	7	46926695	C	0.0531
7p12.3	rs3801081	7:47511161_A/G	7	47511161	G	0.0253
8q24.21	rs7013278	8:128414892_T/C	8	128414892	T	0.0091
8q24.21	rs4313119	8:128571855_G/T	8	128571855	G	0.0518
8q23.3	rs16892766	8:117630683_A/C	8	117630683	C	0.2099
8q23.3	rs6469654	8:117632965_G/C	8	117632965	G	0.0677
8q24.11	rs117079142	8:117790914_C/A	8	117790914	A	0.1139
8q24.21	rs6983267	8:128413305_G/T	8	128413305	G	0.1052
9q22.33	rs34405347	9:101679752_T/G	9	101679752	T	0.0089
9p21.3	rs1537372	9:22103183_G/T	9	22103183	G	0.0120
9q31.3	rs10980628	9:113671403_T/C	9	113671403	C	0.0511
10p14	rs12217641	10:8663875_C/T	10	8663875	C	0.0069
10q24.2	rs10786560	10:101315166_G/A	10	101315166	G	0.0082
10q22.3	rs1250567	10:81046265_T/C	10	81046265	C	0.0470
10p14	rs11255841	10:8739580_T/A	10	8739580	T	0.1064
10q11.23	rs10821907	10:52648454_C/T	10	52648454	C	0.0730
10q22.3	rs704017	10:80819132_A/G	10	80819132	G	0.0765
10q24.2	rs11190164	10:101351704_A/G	10	101351704	G	0.0889
10q25.2	rs12246635	10:114288619_T/C	10	114288619	C	0.0975
10q25.2	rs11196170	10:114722621_G/A	10	114722621	A	0.0527
11q13.4	rs7946853	11:74409077_T/C	11	74409077	C	0.0119
11q22.1	rs55864876	11:100717136_G/A	11	100717136	G	0.0150

Table S1, continued.

Locus	rsID lead variant	Variant	Chr.	Position (Build 37)	Risk allele	Reported beta coefficient risk allele
11q22.1	rs2186607	11:101656397_T/A	11	101656397	T	0.0483
11q13.4	rs61389091	11:74427921_C/T	11	74427921	C	0.1934
1p15.4	rs4450168	11:10286755_A/C	11	10286755	C	0.0413
11q12.2	rs174533	11:61549025_G/A	11	61549025	G	0.0636
11q13.4	rs7121958	11:74280012_T/G	11	74280012	G	0.0780
11q23.1	rs3087967	11:111156836_T/C	11	111156836	T	0.1122
12q13.3	rs4759277	12:57533690_C/A	12	57533690	A	0.0285
12q24.21	rs1427760	12:115100714_T/C	12	115100714	C	0.0424
12p13.32	rs3217874	12:4400808_C/T	12	4400808	T	0.0453
12p13.31	rs10849433	12:6406904_T/C	12	6406904	C	0.0468
12q12	rs11610543	12:43134191_A/G	12	43134191	G	0.0474
12p13.32	rs35808169	12:4368607_T/C	12	4368607	C	0.0890
12p13.32	rs3217810	12:4388271_C/T	12	4388271	T	0.1181
12p13.31	rs2250430	12:6421174_A/T	12	6421174	T	0.0597
12p11.21	rs77969132	12:31594813_C/T	12	31594813	T	0.1583
12q13.12	rs12372718	12:51171090_A/G	12	51171090	G	0.0896
12q24.12	rs597808	12:111973358_A/G	12	111973358	G	0.0737
12q24.21	rs7300312	12:115890922_T/C	12	115890922	C	0.0660
12p13.2	rs2710310	12:12035649_C/T	12	12035649	C	0.0145
13q22.1	rs78341008	13:73791554_T/C	13	73791554	C	0.0109
13q34	rs8000189	13:111075881_C/T	13	111075881	T	0.0473
13q22.1	rs45597035	13:73649152_A/G	13	73649152	A	0.0495
13q22.1	rs1924816	13:73997961_A/G	13	73997961	A	0.0506
13q13.3	rs7333607	13:37462010_A/G	13	37462010	G	0.0758
13q22.3	rs1330889	13:78609615_T/C	13	78609615	C	0.0453
13q13.2	rs377429877	13:34092164_C/T	13	34092164	C	0.0468
14q22.2	rs1951864	14:54369299_G/A	14	54369299	A	0.0059
14q23.1	rs17094983	14:59189361_G/A	14	59189361	G	0.0062
14q23.1	rs8020436	14:59208437_G/A	14	59208437	A	0.0294
14q22.2	rs35107139	14:54419106_A/C	14	54419106	C	0.0912
14q22.2	rs4901473	14:54445157_G/A	14	54445157	G	0.0465
15q23	rs745213	15:68060389_T/G	15	68060389	G	0.0072
15q22.31	rs12594720	15:67007018_C/G	15	67007018	C	0.0246
15q22.33	rs56324967	15:67402824_T/C	15	67402824	C	0.0689
15q13.3	rs17816465	15:33156386_G/A	15	33156386	A	0.0690
15q13.3	rs12708491	15:32992836_G/A	15	32992836	G	0.0464
15q13.3	rs2293581	15:33010736_G/A	15	33010736	A	0.1248
15q26.1	rs7495132	15:91172901_C/T	15	91172901	T	0.0453
16q23.2	rs9930005	16:80043258_C/A	16	80043258	C	0.0061

Table S1, continued.

Locus	rsID lead variant	Variant	Chr.	Position (Build 37)	Risk allele	Reported beta coefficient risk allele
16q24.1	rs12447408	16:86252544_G/A	16	86252544	A	0.0079
16q22.1	rs9924886	16:68743939_A/C	16	68743939	A	0.0550
16q24.1	rs12149163	16:86339315_T/C	16	86339315	T	0.0487
16q24.1	rs62042090	16:86703949_C/T	16	86703949	T	0.0481
17q24.3	rs983318	17:70413253_G/A	17	70413253	A	0.0397
17p13.3	rs73975586	17:814243_A/T	17	814243	A	0.0497
17p12	rs1078643	17:10707241_G/A	17	10707241	A	0.0747
17q25.3	rs75954926	17:81061048_A/G	17	81061048	G	0.0882
17q25.3	rs373585858	17:80394556_G/A	17	80394556	A	0.1103
17p13.3	rs4968127	17:809643_G/A	17	809643	G	0.0514
18q21.1	rs11874392	18:46453156_A/T	18	46453156	A	0.1606
19q13.43	rs73068325	19:59079096_C/T	19	59079096	T	0.0066
19p13.11	rs34797592	19:16417198_C/T	19	16417198	T	0.0824
19q13.11	rs28840750	19:33519927_T/G	19	33519927	T	0.1939
19q13.2	rs1963413	19:41871573_G/A	19	41871573	A	0.0441
19q13.33	rs12979278	19:49218602_C/T	19	49218602	T	0.0293
20q13.33	rs2738783	20:62308612_T/G	20	62308612	T	0.0060
20q13.13	rs6067417	20:48983697_C/T	20	48983697	C	0.0331
20q13.12	rs6031311	20:42666475_C/T	20	42666475	T	0.0362
20q13.13	rs6091189	20:49256285_C/T	20	49256285	T	0.0549
20p12.3	rs994308	20:6603622_C/T	20	6603622	C	0.0626
20p12.3	rs28488	20:6762221_C/T	20	6762221	T	0.0714
20p12.3	rs556532366	20:8568071_C/T	20	8568071	T	0.0715
20p12.3	rs189583	20:6376457_G/C	20	6376457	G	0.0795
20p12.3	rs4813802	20:6699595_T/G	20	6699595	G	0.0819
20p12.3	rs11087784	20:7740976_A/G	20	7740976	G	0.0874
20q13.13	rs6066825	20:47340117_A/G	20	47340117	A	0.0719
20q13.13	rs6063514	20:49055318_C/T	20	49055318	C	0.0547
20q13.32	rs13831	20:57475191_A/G	20	57475191	G	0.0334
20q13.33	rs1741640	20:60932414_T/C	20	60932414	C	0.1146
20q11.22	rs6058093	20:33213196_A/C	20	33213196	C	0.0450

Abbreviations: A, adenine; C, cytosine; G, guanine; T, thymine.

[a] The missing reference single-nucleotide polymorphism was replaced by rs6904092 (linkage disequilibrium, D'=1 and r2=1).

Reference

1.Thomas M, Sakoda LC, Hoffmeister M, et al. Genome-wide Modeling of Polygenic Risk Score in Colorectal Cancer Risk. Am J Hum Genet. Sep 3 2020;107(3):432-444. doi:10.1016/j.ajhg.2020.07.006

Table S2. Characteristics of the study population with complete information on red and processed meat intake

Characteristics	No finding, N (%)	Any neoplasm, N (%)		*p*-value[e]
		Overall	Advanced neoplasm	
Total	4,864	2,427	945	
Sex				<0.0001
Female	2,803 (57.6%)	908 (37.4%)	362 (38.3%)	
Male	2,061 (42.4%)	1,519 (62.6%)	583 (61.7%)	
Age (year, Median (Q^{25}, Q^{75}))	60 (56, 66)	62 (57, 68)	62 (57, 69)	<0.0001
Education (year)				0.0199
<10	2,542 (52.3%)	1,350 (55.6%)	527 (55.8%)	
10-11	1,241 (25.5%)	577 (23.8%)	202 (21.4%)	
>11	1,038 (21.3%)	475 (19.6%)	204 (21.6%)	
BMI (kg/m^2)				<0.0001
<25	1,798 (37.0%)	686 (28.3%)	286 (30.3%)	
25-<30	1,988 (40.9%)	1,129 (46.5%)	421 (44.6%)	
≥30	1,005 (20.7%)	581 (23.9%)	227 (24.0%)	
Smoking status				<0.0001
Never	2,516 (51.7%)	1,033 (42.6%)	366 (38.7%)	
Former	1,728 (35.5%)	927 (38.2%)	361 (38.2%)	
Current	571 (11.7%)	447 (18.4%)	212 (22.4%)	
Alcohol consumption[a]				<0.0001
None	1,268 (26.1%)	506 (20.8%)	204 (21.6%)	
Low	1,827 (37.6%)	817 (33.7%)	289 (30.6%)	
Low-moderate	919 (18.9%)	557 (23.0%)	215 (22.8%)	
Moderate-high	542 (11.1%)	349 (14.4%)	157 (16.6%)	
High	136 (2.8%)	114 (4.7%)	52 (5.5%)	
Physical activity[b]				0.9962
<30 min/day	143 (2.9%)	72 (3.0%)	27 (2.9%)	
≥30 min/day	4,663 (95.9%)	2,321 (95.6%)	905 (95.8%)	
Red meat intake[c]				<0.0001
≤1 time/week	2,272 (46.7%)	977 (40.3%)	379 (40.1%)	
>1 time/week	2,592 (53.3%)	1,450 (59.7%)	566 (59.9%)	
Processed meat intake[c]				<0.0001
≤1 time/week	1,178 (24.2%)	393 (16.2%)	157 (16.6%)	
>1 time/week	3,686 (75.8%)	2,034 (83.8%)	788 (83.4%)	
>1 time/week and <1 time/day	1,928 (39.6%)	1,001 (41.2%)	404 (42.8%)	
≥1 time/day	1,758 (36.1%)	1,033 (42.6%)	384 (40.6%)	
History of HRT[d]	1,112 (22.9%)	332 (13.7%)	123 (13.0%)	0.1185
History of diabetes	443 (9.1%)	295 (12.2%)	117 (12.4%)	0.0001
Family history of CRC	597 (12.3%)	325 (13.4%)	132 (14.0%)	0.1885
Use of NSAIDs	794 (16.3%)	424 (17.5%)	163 (17.2%)	0.2136
History of colonoscopy	1,479 (30.4%)	632 (26.0%)	214 (22.6%)	0.0001
Whole grain intake[c] (<1 time/day)	2,741 (56.4%)	1,472 (60.7%)	570 (60.3%)	0.0002
Fruit intake[c] (<1 time/day)	1,814 (37.3%)	999 (41.2%)	409 (43.3%)	0.0021

Vegetable intake[c] (<1 time/day)	2,225 (45.7%)	1,231 (50.7%)	493 (52.2%)	0.0001
Poultry meat intake[c] (<1 time/week)	1,879 (38.6%)	977 (40.3%)	388 (41.1%)	0.1390

Note: number of missing participants in No finding/Any neoplasm/Advanced neoplasm: education 43/25/12, BMI 73/31/11, smoking status 49/20/6, alcohol consumption 172/84/28, physical activity 58/34/13, history of hormone replacement therapy 33/14/7, history of diabetes 28/8/1, use of NSAIDs 345/176/67, whole grain intake 70/45/25, fruit intake 28/6/5, vegetable intake 10/4/2, poultry meat intake 73/48/16.

[a]Alcoholic consumption in the past 12 months: None: 0 g/day; Low: 0-<12 g/day; Low-moderate: 12-<25 g/day; Moderate-high: 25-<50 g/day; High: ≥ 50 g/day.

[b]Physical activity in the previous 12 months.

[c]Consumption in the previous 12 months.

[d]N (%) was calculated among female participants.

[e]Comparing participants with and without neoplasm.

Abbreviations: BMI, body mass index; CRC, colorectal cancer; HRT, hormone replacement therapy; NSAID, non-steroidal anti-inflammatory drug.

Table S3. Association of red and processed meat intake with colorectal neoplasms risk among participants with complete information on meat intake

	Compared Groups, N(%)[a]		OR(95% CI)[b]	OR(95% CI)[c]
**Red meat intake**				
	**No finding**	**Any neoplasm**		
≤1 time/week	2,272 (46.7)	977 (40.3)	Ref.	Ref.
>1 time/week	2,592 (53.3)	1,450 (59.7)	1.11 (1.00, 1.23)	1.05 (0.95, 1.17)
	**No finding**	**Advanced neoplasm**		
≤1 time/week	2,272 (46.7)	379 (40.1)	Ref.	Ref.
>1 time/week	2,592 (53.3)	566 (59.9)	1.13 (0.97, 1.31)	1.05 (0.90, 1.22)
**Processed meat intake**				
	**No finding**	**Any neoplasm**		
≤1 time/week	1,178 (24.2)	393 (16.2)	Ref.	Ref.
>1 time/week	3,686 (75.8)	2034 (83.8)	1.37 (1.20, 1.57)	1.28 (1.12, 1.46)
>1 time/week and <1 time/day	1,928 (39.6)	1,001 (41.2)	1.35 (1.17, 1.56)	1.28 (1.10, 1.47)
≥1 time/day	1,758 (36.1)	1,033 (42.6)	1.40 (1.21, 1.61)	1.28 (1.10, 1.48)
	**No finding**	**Advanced neoplasm**		
≤1 time/week	1,178 (24.2)	157 (16.6)	Ref.	Ref.
>1 time/week	3,686 (75.8)	788 (83.4)	1.36 (1.12, 1.64)	1.24 (1.02, 1.51)
>1 time/week and <1 time/day	1,928 (39.6)	404 (42.8)	1.38 (1.13, 1.69)	1.30 (1.05, 1.60)
≥1 time/day	1,758 (36.1)	384 (40.6)	1.33 (1.08, 1.64)	1.18 (0.95, 1.47)

[a]Participants with complete information on meat intake regardless of genetic information.

[b]Adjusted for age and sex.

[c]Additionally adjusted for education, smoking status, BMI, physical activity, alcohol consumption, history of hormone replacement therapy, history of diabetes, use of NSAIDs, family history of CRC in a first-degree relative, history of colonoscopy, whole grain intake, fruit intake, vegetable intake and poultry meat intake.

Abbreviations: BMI, body mass index; CI, confidence interval; NSAID, non-steroidal anti-inflammatory drug; OR, odds ratio; Ref., reference.

Supplementary Method

Derivation of Genetic Risk Equivalent (GRE)

The concept of GRE has been introduced in analogy with the well-established concept of risk and rate advancement periods (1). Briefly, consider an analysis based on a multivariable logistic regression:

$$\ln(R) = a + b_1 \cdot M + b_2 \cdot P + \sum_{i=1}^{n} c_i \cdot F_i$$

where ln(R) reflects the log odds of the disease risk and a, b_1, b_2 and c_i (i = 1, . . ., n) refer to the intercept and model parameters for M (meat intake, categorized as 1 for more than 1 time/week group and 0 for the group with less than or equal to 1 time/week), P (PRS percentile according to the distribution of PRS among controls), and F (other covariates), respectively.

GRE equals to the ratio of b_1 and b_2, the estimated coefficients for meat intake and PRS:

$$\text{GRE} = \frac{b_1}{b_2}$$

Thus, the properties of GRE follow from the corresponding properties of b_1 and b_2, which include consistency, asymptotic unbiasedness and normality. Using the delta method, the asymptotic variance of GRE can be derived as:

$$\text{var}(\text{GRE}) = \frac{1}{{b_2}^2}\left[\text{var}\left(b_1\right) - 2\cdot\left(\frac{b_1}{b_2}\right)\cdot\text{cov}\left(b_1, b_2\right) + \left(\frac{b_1}{b_2}\right)^2\cdot\text{var}\left(b_2\right)\right]$$

As the GRE is asymptotically normal, its 95% CIs can be easily calculated using the square root of var(GRE):

$$\text{GRE} \pm 1.96\sqrt{\text{var(GRE)}}$$

Reference:

1. Brenner H, Gefeller O, Greenland S. Risk and rate advancement periods as measures of exposure impact on the occurrence of chronic diseases. Epidemiology 1993 May;4(3):229–36.
